# Progression of AFP SCORE is a Preoperative Predictive Factor of Microvascular Invasion in Selected Patients Meeting Liver Transplantation Criteria for Hepatocellular Carcinoma

**DOI:** 10.3389/ti.2022.10412

**Published:** 2022-03-23

**Authors:** Astrid Herrero, Lucile Boivineau, Gianluca Cassese, Eric Assenat, Benjamin Riviere, Stéphanie Faure, José Ursic Bedoya, Fabrizio Panaro, Boris Guiu, Francis Navarro, Georges-Philippe Pageaux

**Affiliations:** ^1^ Department of Digestive Surgery and Liver Transplantation, Montpellier University Hospital, University of Montpellier, Montpellier, France; ^2^ Liver Transplantation Unit, Department of Hepatology, Montpellier University Hospital, University of Montpellier, Montpellier, France; ^3^ Department of Clinical Medicine and Surgery, Minimally invasive and robotic HPB Surgery Unit, Federico II University, Naples, Italy; ^4^ Department of Digestive Oncology, Montpellier University Hospital, University of Montpellier, Montpellier, France; ^5^ Department of Pathology, Montpellier University Hospital, University of Montpellier, Montpellier, France; ^6^ Department of Digestive Imaging, Montpellier University Hospital, University of Montpellier, Montpellier, France

**Keywords:** hepatocellular carcinoma, microvascular invasion, liver transplantation, recurrence, delta AFP score

## Abstract

Microvascular invasion (MVI) is one of the main prognostic factors of hepatocellular carcinoma (HCC) after liver transplantation (LT), but its occurrence is unpredictable before surgery. The alpha fetoprotein (AFP) model (composite score including size, number, AFP), currently used in France, defines the selection criteria for LT. This study’s aim was to evaluate the preoperative predictive value of AFP SCORE progression on MVI and overall survival during the waiting period for LT. Data regarding LT recipients for HCC from 2007 to 2015 were retrospectively collected from a single institutional database. Among 159 collected cases, 34 patients progressed according to AFP SCORE from diagnosis until LT. MVI was shown to be an independent histopathological prognostic factor according to Cox regression and competing risk analysis in our cohort. AFP SCORE progression was the only preoperative predictive factor of MVI (OR = 10.79 [2.35–49.4]; *p* 0.002). The 5-year overall survival in the progression and no progression groups was 63.9% vs. 86.3%, respectively (*p* = 0.001). Cumulative incidence of HCC recurrence was significantly different between the progression and no progression groups (Sub-HR = 4.89 [CI 2–11.98]). In selected patients, the progression of AFP SCORE during the waiting period can be a useful preoperative tool to predict MVI.

## Graphical Abstract

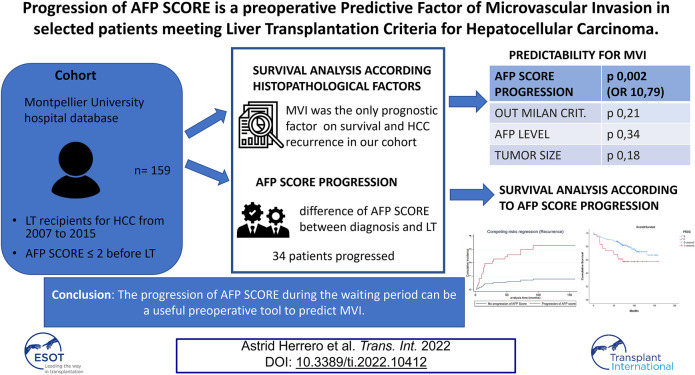

## Introduction

Hepatocellular carcinoma (HCC) is a major health problem worldwide, ranked sixth for cancer incidence and third for cancer-related deaths (1). Liver transplantation (LT) represents the only curative therapy being one of the few tumors treated by organ transplantation when diagnosed at an early stage (2).

Five-year overall survival (OS) ranges from 65 to 80% (3, 4, 5), but it is challenged by two events that cannot be avoided: the waiting period, with the growing risk of dropout (5–10%), and the risk of recurrence (6), highly influenced by the post-transplant immunosuppressive regimen. Due to organ shortage and to the ethical principle of equity, patients with HCC are constantly “competing” with cirrhotic patients, prioritizing patients with more severe disease according to the Model for End-Stage Liver Disease (MELD) score (7, 8, 9).

All these criteria must be integrated before LT for HCC to optimize benefit on survival and limit futile transplants due to tumor recurrence leading to rapid death and graft loss.

Milan criteria, based on tumor size and number of nodules, are considered the benchmark of transplant patients selection, but despite their use, HCC still has a recurrence of 10%–15% (10, 11).

HCC recurrence after LT significantly affects long-term patient survival (12, 13). Microvascular invasion is a well-known risk factor for recurrence, as well as poor differentiation, tumor size, tumor number, and satellite nodules (14, 15, 16). Unfortunately, histological data are only available after LT, and prediction of microvascular invasion before treatment remains one of the main challenges for physicians involved in LT. Alpha fetoprotein (AFP) is the main biomarker that has been shown to predict microvascular invasion, dropout, and recurrence (17, 18, 19). However, only half of patients with HCC have abnormal AFP levels and it cannot be the only variable taken into account (20); previous papers have always focused on cohorts of non-secreting tumors (21).

In France, HCC has become the first indication for liver transplantation, concerning 30% of patients on the waiting list (22). The French Study group for LT reported a new predictive model for HCC recurrence, called AFP SCORE, that was officially endorsed in 2013 by the French organ sharing organization (Agence de la Biomédecine, ABM) (23). The use of an AFP SCORE ≤2 in the last trimester preceding LT has been shown to reduce the risk of HCC recurrence up to 10% (24, 25). Only the last static AFP SCORE value is considered for decision-making in current practice.

While several studies analyzed the interest of dynamic AFP measurements as a possible prediction tool for dropout or post liver transplant recurrence (21), no studies investigating the variation effect on AFP SCORE exist. Yet, being assessed every 3 months during the waiting period by the French organ sharing organization, it could be a relevant and an easy-to-use tool.

The main objective of our study was to evaluate the impact of the AFP SCORE variation during the waiting period to preoperatively predict the microvascular invasion risk in a selected population of LT recipients with a histologically proven AFP SCORE ≤2.

## Patients and Methods

This is a single institution observational retrospective study, conducted according to the Strengthening and the Reporting of Observational Studies in Epidemiology (STROBE) guidelines of the EQUATOR network (26). All consecutive adult recipients who underwent LT for HCC from January 2007 to December 2015 were reviewed. All patients gave their informed consent prior to their inclusion in the study. The study was registered in the institutional review board of the Montpellier University Hospital (N° 2018_IRB-MTP_11-23). The inclusion criteria were defined according to the AFP model (23, 24), in accordance with French national guidelines, considering LT for patients with HCC with an AFP SCORE ≤2 at the last trimester preceding LT. HCC was histologically proven on the native liver. Patients with an AFP SCORE >2 at HCC diagnosis but were down-staged by locoregional therapies to fit transplantation criteria and patients who underwent LT for recurrence after a first liver resection or ablation (salvage transplantation) were also included. The exclusion criteria included presence of cholangiocarcinoma, incidental finding of HCC on the explant, and patients transplanted within 3 months after the diagnosis of HCC.

Data regarding LT recipients’ age, gender, BMI, primary etiology of cirrhosis, and Child Pugh and MELD scores were collected. Tumor characteristics collected at the diagnosis before any treatment were number of nodules, size of the largest nodule, AFP level, the AFP SCORE, and grading according to the Milan criteria. Histopathology data collected on the explant were size of the largest nodule (mm), number of nodules, tumor differentiation according to the WHO classification, microvascular invasion, macrovascular invasion, and satellite nodules (27).

### Management During the Waiting Period and the Follow up

All variables of interest were evaluated every 3 months during the waiting period by CT scan or MRI and blood sample analysis until liver transplantation (based on the national protocol for patients on the waiting list for liver transplantation). Any bridging therapies during the waiting period were decided by the institutional weekly Multi-Disciplinary Team in HCC of the Montpellier University Hospital, in accordance with the European and French guidelines (28, 29). All bridging therapies performed were reported. The delay between HCC diagnosis and the inscription on the waiting list and the delay between the inscription and the liver transplantation was recorded. Over the study follow-up period, the same standard immunosuppressive regimen was followed by LT recipients, consisting of tacrolimus (plus steroids for the first 3–6-month period post-LT) ± mycophenolate mofetil. Follow-up was scheduled every 3 months during the first year after LT, then every 6 months until May 2020. Tumor recurrence was screened performing serum AFP levels and chest and abdominal CT scans or hepatic ultrasounds every 3 months during the 2 first post-operative years, and then twice a year and/or when clinically indicated.

### Definition of AFP SCORE Progression

The AFP SCORE (0–9 points) was calculated depending on largest tumor diameter (≤3 cm = 0 points, 3–6 cm = 1 point, >6 cm = 4 points), number of HCC nodules (1–3 nodules = 0 points, ≥4 nodules = 2 points), and pre-LT AFP levels ng/ml (≤100 = 0 points, 101–1,000 = 2 points, and >1,000 = 3 points) (23).

The variation of AFP SCORE was calculated from the difference between AFP SCORE at the diagnosis of HCC and AFP SCORE 3 months before LT, regardless of pre-transplant therapy (radiofrequencies, chemo-embolization, or others), as it is part of the natural history of HCC patients on the waiting list. Patients were classified into two groups: progression (ΔAFP ≥ +1) and non-progression (Δ < 1).

### Endpoints

The primary outcome was the preoperative prediction of MVI on the explant. MVI was defined as the presence of tumor cells in portal veins, in large capsule vessels, or in a vascular space lined by endothelial cells on microscopy. Pathological specimen were evaluated on 5 mm slices, observed by two expert pathologists, blinded from clinical data (30).

Secondary outcomes were OS and HCC recurrence risk after LT according to AFP SCORE progression.

### Statistical Analysis

The categorical data were described by frequencies and percentages, whereas continuous data were described by mean ± standard deviation (SD) or median ± interquartile range (IQR) depending on whether or not they showed a normal distribution. Categorical variables were compared by using the *χ* ^2^ test or Fisher exact test, while continuous variables were compared by applying Student’s t-test or Mann-Whitney test, when appropriate. Median follow-up (and 95% CI) was computed using the reverse Kaplan-Meier method. Overall and disease-free survival were calculated using the Kaplan-Meier method. First, we aimed to confirm the prognostic impact of MVI on OS through a Cox regression analysis incorporating histopathological data determined from the native liver. Secondly, predictive factors associated with MVI were identified using uni- and multivariate logistic regression models. Owing to the relatively limited number of events, relevant variables with a *p* value of less than 0.1 were selected for multivariate analysis via a backward procedure, and an internal validation of the model was performed with 150 bootstrap samples to prevent overfitting (cf Supplementary Material) (31). In addition, the area under the ROC (AUROC) curve was computed to capture the predicting ability of the model. Finally, OS was compared in patients with vs. without AFP SCORE progression, using a log-rank test. HCC recurrence after LT was analyzed in a competing risks framework with HCC recurrence and death as competing events. Cumulative incidence curves for HCC recurrence using Fine-Gray proportional sub-distribution hazards models according to the AFP SCORE progression were performed. Log-linearity was checked and continuous variables were transformed whenever necessary (32).

All analyses were performed with the Stata software, version 17 (Stata Corporation, College Station, TX, United States). A *p*-value < 0.05 was considered significant.

## Results

### Patient and Tumor Characteristics

**FIGURE 1:**
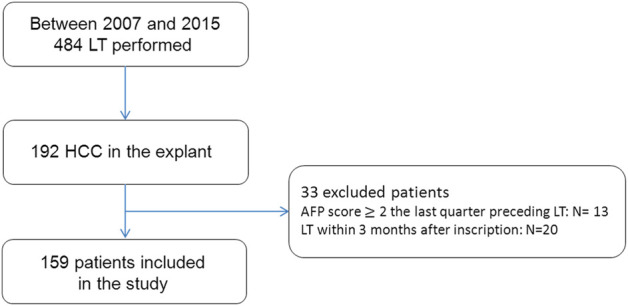
Flow chart of the study population.

From 2007 to 2015, among 484 liver transplantations performed in our hospital, 192 patients presented with HCC on the native liver and 159 patients met the inclusion criteria (Figure 1).

Liver transplantation was performed using full grafts or partial grafts from a split procedure (*n* = 2) from deceased donors. At the diagnosis, the tumor number was 1 in 45% of patients (*n* = 73), 2 in 28% of patients (*n* = 44), and ≥3 in 27% of them (*n* = 42). Median tumor size was 18.5 mm [±17]. The median value of AFP level was 6 ng/ml (range:1–1,584 ng/ml), while AFP SCORE was 0 in 70% of patients (*n* = 111), 1 in 17% (*n* = 27), 2 in 8% (*n* = 13), and ≥3 in 5% of patients (*n* = 8). All patient and tumor characteristics are detailed in Table 1. The mean time on the waiting list was 7.32 months (±5.75). Overall, 119 patients (75%) received one or more bridging therapies to control the disease during the waiting period, according to the institutional multidisciplinary team indications (as detailed in the Supplementary Material).

**TABLE 1:** Comparative analysis according to the AFP SCORE progression during the waiting period.

Characteristics	Total	Progression	No progression	*p*-value
	(*n* = 159)	(*n* = 34)	(*n* = 125)	
Age, mean (range)	57.9 (39–72)	59.3 (46–69)	57.5 (39–72)	0.18
Sex, male/Female	136/23	31/7	105/16	0.85
BMI, kg/m^2^ median (IQR)	26 (±6)	26 (±6)	26 (±7)	0.58
Etiology of cirrhosis (%)				
Hepatitis C	55 (34)	6 (18)	49 (40)	0.01
Hepatitis B	10 (6)	2 (6)	8 (6.5)	0.91
Alcohol	75 (47)	21 (62)	54 (43)	0.055
NASH	11 (7)	4 (12)	7 (5.6)	0.20
Other	8 (6)	1 (2)	7 (5.6)	0.52
MELD score, Median (IQR)	11 (±9)	10 (±5.25)	12 (±9)	0.13
MELD >30 (%)	2 (1)	0	2 (99)	0.45
MELD <30 (%)	157 (99)	34 (100)	123 (1)	
Child- Pugh score (%)				
A	79 (49)	19 (56)	60 (49)	0.49
B	52 (32)	10 (30)	42 (34)	0.64
C	26 (19)	5 (14)	21 (17)	0.61
Time on waiting list, mean (SD)	7.32 (±5.75)	7.85 (±4.95)	7.18 (±5.9)	0.50
Pre-LT tumor treatment (%)	119 (78)	24 (64)	95 (76)	0.51
Tumor number, (%)				
1	73 (45)	19 (50)	67 (50)	0.81
2	44 (28)	8 (21)	36 (27)	0.55
≥3	42 (27)	11 (29)	31 (23)	0.37
Tumor size, mm, Median (IQR)	18.5 (±17)	24 (±11)	22 (±13)	0.68
Diameter of largest nodule (%)				
≤30 mm	124 (78)	95 (76)	29 (85)	0.28
30–60 mm	32 (20)	27 (22)	5 (15)	0.37
>60 mm	3	3 (2)	0	
AFP at diagnosis, ng/ml, median (range)	6 [1–1,584]	5.95 (2–607)	6 (1–1,584)	0.27
AFP at diagnosis, ng/ml				
≤100	153 (96.5)	120 (96)	33 (97)	0.77
100–1,000	5 (3)	4 (3)	1	0.93
>1,000	1 (0.5)	1	0	
AFP SCORE at diagnosis				
0	111 (70)	28 (82)	83 (66)	0.07
1	27 (17)	5 (15)	22 (18)	0.69
2	13 (8)	1 (3)	12 (10)	0.20
≥3	8 (5)	0	8 (6)	

Pathological examination of the explanted liver showed that the median tumor size was 30 mm (±20 mm). The HCC nodule was solitary in 55 patients (22%), while 64 LT recipients had three or more lesions (40%), and 89 patients (56%) were within the Milan criteria. Among the 70 LT recipients (44%) beyond the Milan criteria, histological analysis showed a tumor size >50 mm in six patients, four or more nodules in 35 patients; 29 patients had less than three nodules but with a tumor size >30 mm. According to the WHO classification, the tumor was well, moderate, or poorly differentiated in 28, 62, and 10% of patients, respectively. MVI was found in 31 (19.5%) explants. Among them, 14 presented satellite nodules. The histopathological results are shown in Table 2.

**TABLE 2:** Histopathological features on surgical specimens after liver explant.

MVI (%)	31 (20)
Macrovascular invasion (%)	4 (2.5)
Satellite nodules (%)	19 (11.9%)
Median tumor size (IQR)	30 (20)
Median tumor number (IQR)	2 (2)
Poor differentiation (%)	15 (9.4)

MVI, microvascular invasion; IQR, inter-quartile range; poor differentiation, G3 sec. Edmonson.

### Survival Analysis According to Post-Operative Histopathologic Factors on the Native Liver

After a median follow-up of 94 months [95% CI: 83–105], a total of 43 patients died (28.1%). Among them, 29 did not recur. HCC recurrence was observed in 19 patients (12%) within a median delay of 13 (range 2–92) months, and 14 of them died after the recurrence (73.6%). The 90-day post-operative mortality was 2.5% (4 patients). Three- and 5-year OS was 86.1% and 81.5%.

Cox regression analysis showed MVI as the only histopathological prognostic factor (among tumor differentiation, tumor size, number of nodules, and the presence of satellite nodules) of overall survival (HR 3.85 [95% CI 1.98–7.49]; *p* < 0.0001) (Table 3). Competing risk analysis for HCC recurrence identified MVI as an independent prognostic factor (SHR 8.11 [CI 3.13–20.96]; *p* < 0.0001) (Table 4).

**TABLE 3:** Cox regression model for overall survival, univariate analysis.

Cox regression model for overall survival according to histopathological features		
	HR (CI 95%)	*p*-value
MVI	**3.85 (1.98–7.49)**	**0.000**
Poor differentiation	0.89 (0.34–2.29)	0.815
Satellite nodules	0.85 (0.31–2.30)	0.460
Tumor size >30 mm	1.63 (0.85–3.14)	0.139
Tumor nodule >3	1.31 (0.63–2.69)	0.460

Bold values represent statistically significant results

MVI, microvascular invasion; SHR, sub-distribution hazard ratio; CI, confidence interval.

**TABLE 4:** competing risk analysis for HCC recurrence according to post-operative histopathological factors.

Competing risk analysis for HCC recurrence		
	*p*-value multivariate SHR [CI]	*p*-value univariate
MVI	**0.000 8.11 [CI 3.13–20.96]**	**0.000**
Poor differentiation	0.403	**0.056**
Satellite nodules		0.72
Tumor size >30 mm		0.47
Tumor nodule >3		0.08

Bold values represent statistically significant results

MVI, microvascular invasion; SHR, sub-distribution hazard ratio; CI, confidence interval.

### AFP SCORE Variation During the Waiting Period

According to the AFP SCORE, 34 LT recipients showed progression during the waiting period.

Progressed and non-progressed patients were statistically comparable with respect to all patient and tumor characteristics, as well as time on the waiting list and bridging therapies. The only statistically significative difference was in cirrhosis etiology where the incidence of progression was lower in the HCV group (Table 1).

### Primary Outcome: Preoperative Predictive Risk Factors on Microvascular Invasion

Tumor size larger than 30 mm, beyond Milan criteria, AFP value pre-LT, and AFP SCORE progression were associated (i.e., *p*-value <0.10) with MVI in univariate analysis (Table 5). When tested with multivariate analysis, the AFP SCORE progression was the only independent preoperative risk factor of MVI (OR = 10.79 [95% CI = 2.35–49.4]; *p* 0.002). A 0.74 AUROC confirmed the good predictive ability of the multivariate model.

**TABLE 5:** Multivariate logistic regression analysis for prediction of MVI in patients undergoing LT for HCC.

	Tot	MVI	No MVI	Univariate analysis		Multivariate analysis	
				Odds ratio (95% CI)	*p*-value	Odds ratio (95% CI)	*p*-value
Overall *n* (%)	159 (100)	31 (19.5)	128 (80.5)				
Age >60 (%)	81 (50.9)	14 (17.3)	67 (82.7)	0.74 (0.34–1.64)	0.47		
BMI >30 (%)	37 (23.2)	27 (73)	10 (27)	1.78 (0.74–4.22)	0.17		
Cirrhosis etiology (%)							
HCV	55 (34)	6 (11)	49 (89)	0.38 (0.14–1.02)	0.10		
HBV	10 (6)	3 (30)	7 (70)	1.85 (0.45–7.61)	0.28		
Alcoholic	75 (47)	14 (18.6)	61 (81.4)	0.90 (0.41–1.98)	0.74		
NASH	11 (7)	2 (18.2)	9 (81.8)	0.91 (0.18–4.44)	0.95		
Other	8 (6)	3 (37.5)	5 (62.5)	2.63 (0.59–11.6)	0.13		
Waiting time, mean (SD)	6.78 (±5.75)	7.07 (±5.77)	7.38 (±5.79)	1.003 (0.96–1.04)	0.78		
No treatments during waiting time (%)	40 (25.1)	10 (25)	30 (75)	1.55 (0.66–3.66)	0.31		
Tumor size >30 mm (%)	17 (10.7)	6 (35)	11 (65)	**2.55 (0.86–7.55)**	**0.08**	1.49 (0.39–5.7)	0.18
Tumor nodules pre-LT ≥3 (%)	53 (33.3)	10 (19)	43 (81)	0.94 (0.40–2.17)	0.30		
Child- Turgot- Pugh (%)							
A	79 (49.6)	18 (22.7)	61 (77.7)	1.52 (0.68–3.36)	0.29		
B	52 (32.7)	7 (13.4)	45 (86.6)	0.53 (0.21–1.34)	0.18		
C	28 (17.6)	6 (21.5)	22 (78.5)	1.15 (0.42–3.15)	0.77		
MELD, median (IQR)	11 (±9)	11 (±6)	11 (±9)	0.97 (0.90–1.04)	0.42		
AFP pre-LT ng/ml, median (range)	6.65 (1–1,170)	12.7 (1.2–1,170)	5.3 (1–373)	**1.02 (0.99–1.03)**	**0.07**	1.03 (0.98–1.05)	0.34
Within Milan criteria (%)	114 (71.6)	19 (16.6)	98 (83.4)	**0.48 (0.21–1.11)**	**0.08**	0.17 (0.01–1.18)	0.21
Delta AFP SCORE progression	34 (21.3)	18 (53)	16 (47)	**9.69 (3.9–23.4)**	**<0.0001**	**10,79 (CI = 2.35–49.4)**	**0.002**

Bold values represent statistically significant results

BMI, body mass index; HCV, hepatitis C virus; HBV, hepatitis B virus; NASH, non-alcoholic steatohepatitis; LT, liver transplantation; MELD, model for end stage liver Disease; AFP, alpha fetoprotein; MVI, microvascular invasion. OR, odds ratio; CI, confidence interval. Variables with *p*-value <0.10 underwent multivariate analysis.

### Secondary Outcomes: Survival Analysis and Recurrence According to Preoperative AFP SCORE Progression

**FIGURE 2:**
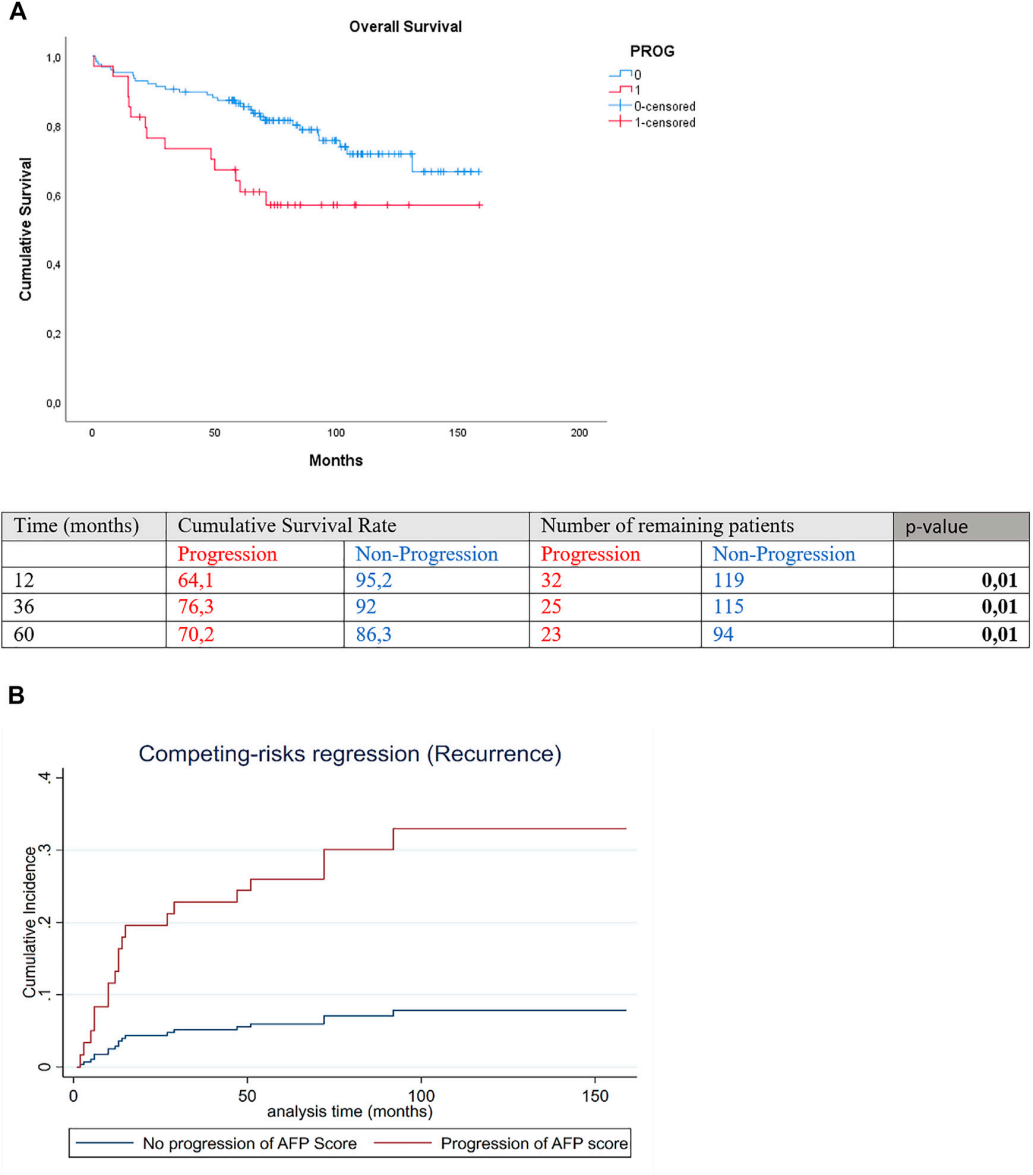
**(A)** Overall survival and **(B)** competing risk regression for HCC recurrence of patients with and without delta AFP SCORE progression.

Three-year and 5-year overall survival was significantly lower in the progression than the non-progression group [(3-year OS 73.2% vs. 89.6%, 5-year OS 63.9% vs. 86.3%; *p* 0.01] (Figure 2A). Cumulative incidence of recurrence significantly differed between the groups of progression and no progression in AFP SCORE (SHR = 4.89 [CI 2–11.98]; *p* = 0.001 (Figure 2B).

## Discussion

In the present study, for the first time in literature, we analyzed the predictability of AFP SCORE progression on MVI in a homogeneous population of LT recipients with histologically proven HCC who had AFP SCORE ≤2. Our results showed that AFP SCORE progression, for those on the waiting list for liver transplantation, was the only preoperative factor that enabled prediction of MVI. In contrast, the absolute value of AFP, Milan criteria, number of tumor nodules, and tumor size were not associated with MVI, emphasizing the need to use a composite score as defined by the AFP model.

Furthermore, variation of the AFP SCORE can be easily calculated in clinical practice and could be a relevant preoperative tool for predicting tumor aggressiveness and other related outcomes.

Notably, our patient cohort had already undergone a stringent selection process, thus, a low rate of recurrence was expected. Despite this, 19 recurrences were observed and among them 14 died shortly after. In both overall survival and HCC recurrence, MVI was shown to be a strong prognostic factor in our study population, with HR greater than 3. Despite the strict cohort selection, the augmentation of AFP SCORE by one point, even from 0 to 1—expected to be an irrelevant variation—was shown to enable prediction of MVI, with OR greater than 10.

These results could lead to an optimization of pre- or post-transplantation strategies in terms of prevention and surveillance, enabling adapted treatment strategies, radiological monitoring of patients on waiting lists, and adjustment of immunosuppressive therapy after LT.

The downstaging of the tumor burden in HCC patients awaiting transplantation has been widely shown to be advantageous in terms of survival, both in patients still on waiting lists and in those who dropped out of the criteria (33, 34, 35). Therefore, having a dynamic tool for identifying patients at high risk of MVI while in the waiting period could further identify a subgroup of candidates that could benefit from a different strategy. We suggest that further studies should explore this direction.

Several studies have reported that baseline or follow-up AFP levels are correlated with survival and/or tumor recurrence (36, 37). However, increased AFP levels are inconstant in HCC patients, with only 30%–40% of patients having abnormal values (38). Furthermore, previous studies on the topic have always selected the study population by eliminating non-secreting tumors (21). In contrast, our study included all patients transplanted for HCC who had an AFP SCORE ≤2, regardless of the levels of AFP and other parameters.

In our study, 93% of patients had an AFP level <100 ng/ml, with the median value being 6 ng/ml, which for some reason challenged the tools used to select the correct follow-up strategy. In such circumstances, AFP SCORE progression could identify patients who would benefit from a stricter follow-up. Eventually, contrast-enhanced (18)F-choline or (11)C-choline PET/CT could be useful, alone or combined with (18F)-FDG PET/CT (39, 40).

Actually, in current practice, we do not dispose of any specific tool that can predict MVI preoperatively. The originality of our study is that it demonstrates the utility of a simple preoperative dynamic score evaluation that can strongly predict MVI without performing a biopsy or radiological exam. Previous studies demonstrated the potential utility of MRI or circulating cell free DNA (cfDNA) as dynamic preoperative biomarkers. These biomarkers were found to be independent predictors of MVI (41, 42). However, cfDNA use is not widespread, as it is expensive and difficult to perform regularly in all centers due to the technical procedures necessary for genetic analysis. In contrast, AFP SCORE can be measured easily and without additional cost, as no additional exams are needed. Future studies exploring the association between cfDNA and the dynamic assessment of AFP SCORE may provide physicians with an effective tool and consequently help guide the selection of individualized therapies or treatment monitoring before radiologic and/or biologic progression.

Finally, the parameter of AFP SCORE progression can be integrated into a new predictive score for identifying the risk of tumor recurrence. The interest in creating, validating, and developing such a model is demonstrated by the increasing number of interesting similar papers (43, 44, 45). At the same time, previous efforts have not enabled identification of the best model. We believe that AFP progression could be associated with parameters related to the total tumor burden and tumor aggressiveness (pre-LT AFP levels, total tumor volume). All previous hypotheses require prospective studies on larger populations to be corroborated, but we believe that they must be considered in light of the importance of HCC recurrence in LT recipients and the relevance of MVI to recurrence and survival (46).

Our study’s limitations include its retrospective design and relatively small number of events. Therefore, further large-scale, multicenter studies are needed. Another limitation is its use of the AFP SCORE, which at the moment is widely used only in France. Nevertheless, the strengths of our study include its long median follow-up of 94 months, its monocentric character, which ensured the homogeneous management of all patients using the same surgical and medical teams, and its selection of a patient cohort with no known preoperative MVI or recurrence factors.

To conclude, this study highlights the potentially high relevance of AFP SCORE progression as a simple, dynamic, preoperative predictive factor for MVI in patients undergoing LT for HCC. These findings could lead LT units to adopt new strategies before or after LT to optimize the management of such subgroups of patients.

## Data Availability

The original contributions presented in the study are included in the article/Supplementary Material, further inquiries can be directed to the corresponding author.

## Abbreviations

AFP, alpha fetoprotein; DFS, disease-free survival; HCC, hepatocellular carcinoma; LT, liver transplantation; MVI, microvascular invasion; OS, overall survival.
