# Supplementary material for: Progression of AFP SCORE is a Preoperative Predictive Factor of Microvascular Invasion in Selected Patients Meeting Liver Transplantation Criteria for Hepatocellular Carcinoma
Source: Transpl Int. 2022 Mar 23;35:10412. doi: 10.3389/ti.2022.10412 (PMC8983829; doi:10.3389/ti.2022.10412)
Supplement: Supplementary file 1 [file DataSheet1.docx]

| **Supplementary material 1. Management on waiting list and pre-transplantation tumor treatment** | | | |
| --- | --- | --- | --- |
| ***Variable*** | | ***Patients (n=159); n (%)*** | |
| Time from diagnosis to inscription on waiting list, median IQR) | | | 5 [3-58] |
| Time on waiting list, median (range) | | | 6,7 [3-34,5] |
| Pre-LT tumor treatment (%) | | | 119 (75%) |
| Number of pre-LT treatment | | |  |
|  | 1 | | 54 (45%) |
|  | 2 | | 42 (35%) |
|  | 3 | | 13 (8%) |
|  | 4+ | | 4 (2,5%) |
| Type of pre-LT treatment | | |  |
|  | Trans Arterial Chemo-Embolization | | 78 (49%) |
|  | Percutaneous ethanol ablation | | 3 (2%) |
|  | Percutaneous thermal ablation | | 56 (35%) |
|  | Liver resection | | 8 (7%) |
|  | Chemotherapy | | 6 (4%) |

**Supplementary material 2. Bootstrapping for the internal validation of the model to predict MVI. Number of observations:150.**

|  | Odds Ratio | p-value | [95% Conf. Interval] |
| --- | --- | --- | --- |
| AFP score progression | 10.79 | **0.011** | 1.71 - 68.02 |
| Out from Milan criteria | 0.39 | 0.29 | 0.067 - 2.28 |
| Afp value | 0.33 | 0.378 | 0.029 - 3.79 |
| Tumor size > 30mm | 1.01 | 0.99 | 0.13 - 7.67 |
